# Effects of athletic tape on orofacial pain and jaw movements after 24 hours of use: a randomized clinical trial

**DOI:** 10.1590/2317-1782/20242023066en

**Published:** 2024-05-27

**Authors:** Marília dos Santos Faria, Gabriela Carolina Silva Teodoro, Júlia Ana Soares Silva, Tatyana Meneses Emérito, Andréa Rodrigues Motta, Mariana Souza Amaral, Renata Maria Moreira Moraes Furlan

**Affiliations:** 1 Curso de graduação em Fonoaudiologia, Faculdade de Medicina, Universidade Federal de Minas Gerais – UFMG - Belo Horizonte (MG), Brasil.; 2 Curso de graduação em Fonoaudiologia, Centro Universitário Uninovafapi – UNINOVAFAPI - Teresina (PI) - Brasil.; 3 Departamento de Fonoaudiologia, Faculdade de Medicina, Universidade Federal de Minas Gerais – UFMG - Belo Horizonte (MG), Brasil.; 4 Programa de Pós-graduação em Ciências Fonoaudiológicas, Universidade Federal de Minas Gerais – UFMG - Belo Horizonte (MG), Brasil.

**Keywords:** Atlhetic Tape, Pain, Facial Pain, Temporomandibular Joint Disorders, Masticatory Muscles

## Abstract

**Purpose:**

To analyze the sensation of pain and the range of mandibular movements of adult individuals with temporomandibular disorder, before and after the application of the athletic tape.

**Method:**

This is a double-blind randomized clinical trial, in which 22 adults with temporomandibular disorder participated, randomly allocated into two groups, with group A comprising 10 women and one man (mean age 28.2±8.3 years) and group B comprising nine women and two men (mean age 26.2±3.9 years). Group A was submitted to the application of the athletic tape on the masseter with 40% stretch and the group B to the application of the athletic tape on the masseter without stretching. All participants underwent the application of the Diagnostic Criteria for Temporomandibular Disorders (DC/TMD). Pain threshold assessment was performed using an algometer to apply pressure to measurement points. The measurement of mandibular movements was performed using a caliper. The athletic tape was glued using the I technique, with a fixed point over the insertion and a movable point over the origin of the masseter muscle. Participants remained with the athletic tape for 24 hours and were re-evaluated.

**Results:**

There was pain relief in the group A in the temporomandibular joint on the right and at the origin of the masseter on the left. The group B showed a reduction in pain in the left anterior temporal region. No differences were found in mandibular movements after intervention, as well as no difference was found in the comparison by groups.

**Conclusion:**

The use of the athletic tape over the masseter muscle, with stretching, for 24 hours produced relief from the sensation of pain, on the origin of the right masseter and in the right temporomandibular joint, and, without stretching, in the left anterior temporal muscle. There was no difference in the range of mandibular movements.

## INTRODUCTION

Orofacial pain is known in the literature as an umbrella term that encompasses painful sensations in the face and mouth^(1)^. It may be due to temporomandibular disorder (TMD), disorders of dentoalveolar and anatomically related structures, injury or disease of the cranial nerves, orofacial pain with manifestations similar to primary headaches, and idiopathic orofacial pain^(1)^.

TMD comprises musculoskeletal and neuromuscular conditions that include dysfunction of the temporomandibular joint (TMJ) and all related muscles and tissues, in which changes in mastication, swallowing, and speech functions and acute or persistent pain are common symptoms^(2)^. Furthermore, TMD is directly related to the movements and functional activities of the mastication muscles and is commonly evidenced by a reduced range of mandibular movements^(3)^. Its etiology is multifactorial, containing elements such as oral habits, trauma, malocclusion, postural changes, stress, and anxiety^(3,4)^. Studies have demonstrated a higher prevalence in adult women, and females are twice as likely to develop TMD; other aspects such as age, ethnicity, and psychosocial factors should also be considered^(5)^.

TMD therapy have been using various techniques, such as active relaxation exercises^(6)^, stretching^(7)^, massage^(8)^, occlusal stabilization^(8)^, health education, short-wave application^(9)^, and so forth. Recently, the application of athletic tape has been recommended^(10-12)^, with excellent results in pain relief, muscle relaxation, strengthening weakened muscles, and improving blood and lymphatic circulation^(12)^.

This tape was created in 1970 in Japan by Kenzo Kase, a Japanese chiropractor, to assist athletes in the treatment of injuries and rehabilitation of musculoskeletal disorders^(13-15)^. Since then, it has been used by different professionals such as occupational, physical, and speech-language-hearing therapists for muscle rehabilitation or stimulation.

The athletic tape is made of twisted cotton involving elastane micro threads, without added medication, which adheres to human skin and can be stretched for a long time^(16)^. It can be applied with different stretches (0% to 100%) for dermal, muscular, joint, and lymphatic purposes, including increased skin sensitivity, muscular excitation or inhibition, increased blood and lymphatic circulation, and reduced pain^(16)^. Recent studies have shown that the use of this tape provides analgesic effects^(17)^, easing muscle pain^(18)^, increasing mouth opening^(18)^, and, associated with mastication muscle exercises, increasing TMJ mobility^(19)^.

Emérito^(14)^ carried out a bibliographic study on the use of athletic tape as a therapeutic resource in oral motor therapy and concluded that the method is effective in treating orofacial changes and dysfunctions, which makes it an alternative for professionals involved in this treatment.

A systematic literature review with meta-analysis^(12)^ verified the effect of applying athletic tape on masticatory muscles in relieving pain, compared to other interventions, in individuals with TMD. The conclusion states that its use significantly reduces pain in the first week.

The number of studies on the use of athletic tape has increased. However, the literature on its use for TMD and orofacial pain is still limited, and the effects of greater or lesser tape stretching are unknown. Moreover, no studies were found that evaluated the immediate effects of the tape; rather, they addressed effects over a longer period, such as 5 consecutive days ^(17)^, 35 days^(18)^, and 6 weeks^(19)^. Hence, it must be verified whether isolated applications for a short period are enough to have any effect.

Thus, this study aimed to analyze the sensation of pain and the range of mandibular movements of adults with TMD before and after applying athletic tape for 24 hours. It is hypothesized that using the tape on the masseter muscle provides, as verified with the perceived sensory effect, a mandibular posture free from clenching and, consequently, relief from the sensation of orofacial pain in the masseter and temporal jaw elevator muscles and an increase in mouth opening and jaw lateralization and protrusion; and that applying the tape with stretching produces greater effects than applying it without stretching.

## METHOD

This is a double-blind randomized clinical trial with 22 students with TMD from the Medical School of the Federal University of Minas Gerais (UFMG), characterizing it as a convenience sample. Data were collected from April to July 2022 at the Speech-Language-Hearing Functional Health Observatory at the UFMG Medical School. The study followed the CONSORT Reporting Guidelines^(20)^, was approved by the Research Ethics Committee under evaluation report number 4.329.360 (CAAE 36777220.7.0000.5149), and was registered on the REBEC platform under number RBR-46cmrsb. All study participants signed an informed consent form, agreeing with the terms of the research.

The research was announced on the UFMG campus and via online platforms to select participants. The study included individuals over 18 years old, of both sexes, with TMD, and excluded individuals with no orofacial pain on palpation, with wounds in the area where the athletic tape would be applied, a history of allergy to the use of tapes, facial malformation, neurological changes, or neurodegenerative disease, who were taking analgesics, muscle relaxants, or anti-inflammatories, and who did not remain with the tape for the set time.

All participants filled out a form with questions on orofacial complaints, mastication, orofacial pain, allergic history, medication use, and neurological diagnosis to assess eligibility criteria. In the first session, researcher 1 surveyed their medical history, applied the Diagnostic Criteria for Temporomandibular Disorders (DC/TMD)^(21)^, and recorded the assessment of pain intensity on palpation and measurement of mouth opening and jaw protrusion and right and left lateralization.

The pain was assessed with an algometer (Brand Kilter, model FM-207), which applies known pressures at different points on the face. Hence, 0.5 kg of pressure was applied to the TMJ and 1 kg to the masseter (origin, middle, and insertion) and temporal muscle (middle, anterior, and posterior). Then, the participant was asked to report their level of pain on a scale from 0 to 10, in which 0 indicated no pain and 10 the maximum pain possible^(21)^.

Subsequently, evaluator 1 provided a visual model of the mandibular movements of mouth opening and jaw lateralization and protrusion and asked the volunteer to repeat them for measurement with a caliper (Digimess^®^, São Paulo). The following measurements were recorded: overbite, maximum active interincisal distance, and right and left mandibular laterality, as described in the DC^(21)^.

Three measurements were taken to assess mouth opening, namely: painless opening, unassisted maximum opening, and assisted opening. In all measurements, the maximum mouth opening was the distance from the incisal edge of the upper to the lower incisor teeth. First, the participant was asked to open the mouth as much as possible without pain, then as much as possible regardless of pain, and lastly as much as possible with the researcher’s manual assistance, exerting force on the jaw in the direction of opening^(21)^. The overbite measurement was added to the mouth opening measurement.

The participant was asked to move the mandible to the right as much as possible to assess mandibular laterality, measuring the horizontal distance between the dental midlines of the lower and upper incisors^(22)^. The same procedure was used for the opposite side, measuring the midline displacement. When the upper and lower midlines did not coincide (distance greater than 1 mm), the value and direction of the discrepancy were noted. The upper midline was considered as a reference, and the discrepancy value was subtracted from the ipsilateral lateralization and added to the contralateral lateralization.

Jaw protrusion was measured as the horizontal distance from the teeth in occlusion between the medial region of the buccal surface of the lower incisors and the incisal edge of the upper incisors. Next, participants were asked to protrude their jaw, repeating the measurement, based on the distance between the buccal surface of the lower central incisors and the incisal edge of the upper incisors, which were added together to obtain the distance covered by the protruded jaw.

Three measurements were taken in each of these situations. The first one was disregarded, and only the measurement with the highest value was considered.

To apply the athletic tape, researcher 2 allocated participants randomly into groups A and B by drawing. Group A had 11 individuals (10 women and one man), with a mean age of 28.2±8.3 years, and group B had 11 individuals (nine women and two men), with a mean age of 26.2±3.9 years.

Group A had the athletic tape applied over the masseter muscle with a 40% stretch^(18)^, while Group B had it applied over the masseter muscle without stretching, characterizing it as the control group. Researcher 2 applied the tape manufactured by Kinésio Tmax (approved by the Brazilian National Health Surveillance Agency under registration 10410130023) over the masseter muscle using the “I” cut, with an initial anchor point at the mandibular angle^(13)^. The tape was 2.5 cm wide, and its length was defined according to each participant’s anatomical characteristics.

When applied without stretching, the length of the tape was each participant’s measure from the zygomatic arch to the mandibular angle. For an application with 40% stretch, the tape length had to be shorter and was calculated specifically for each participant. The increased length at 100% stretch (delta B) was obtained from the difference between the tape length cut stretched to 100% and the length of this cut without stretching. Then, by cross-multiplication, the original tape length was calculated so that the stretch was only 40% of delta B.

**Figure 1:**
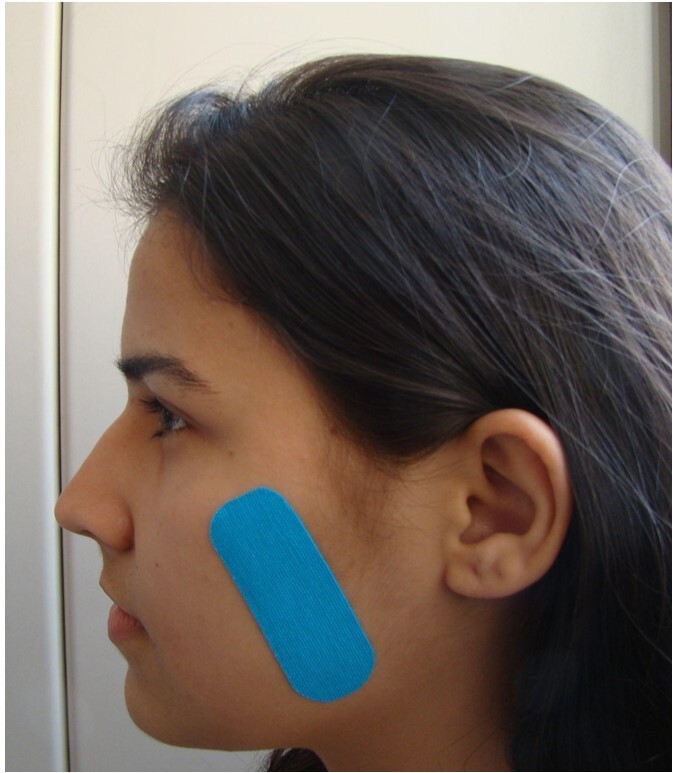
Athletic tape applied over the masseter muscle

The skin region over the masseter muscle was cleaned with gauze soaked in 70% alcohol. Next, researcher 2 measured the masseter muscle from its origin (zygomatic arch) to its insertion (mandibular angle), with a measurement scale provided on the back of the tape. After cutting it, the tape corners were rounded for better fixation and the therapeutic zone and anchors were marked by folding the tape. It was attached from the fixed point to the mobile point (Figure 1). To this end, it was initially anchored (fixed point) at the mandibular angle; then, the participant was asked to open their mouth (maximum opening), and the tape was stretched^(18)^ in group A to attach it to the therapeutic zone. Group B underwent the same procedure, but the tape was not stretched. It was applied bilaterally to all participants, who remained with the tape for 24 hours, without changing their daily routine.

Researcher 1 (responsible for assessing the participants) was not present in the collection room when the tape was applied, being blind as to which group each participant belonged. Likewise, researcher 2 (responsible for allocating participants to groups and applying the tape) was not present when participants were assessed. Participants were also blind to the group to which they were allocated.

After 24 hours, the volunteers returned for the second session, in which researcher 1 removed the athletic tape and measured the level of pain (with an algometer), the mouth opening, and the jaw lateralization and protrusion. Thus, in both groups, the researcher who assessed them was always different from the one who applied the tape, and the former did not know which group the individual belonged to.

After the reassessment, the participants answered a questionnaire to verify their perception of tape use. The questionnaire was based on the study by Silva et al.^(16)^, with five close-ended yes/no questions to check the possible sensations caused by its use, namely: discomfort, itching, relaxation, pain relief, and improved mobility.

The study response variables were the intensity of orofacial pain and range of mandibular movements. They were compared at assessment and reassessment for each group. The explanatory variables were the moment (pre-intervention vs. post-intervention) and tape stretching (group A vs. group B). The data were recorded in a Microsoft Excel spreadsheet and subsequently analyzed with measures of central tendency and dispersion for continuous variables and frequency distribution for categorical ones. The frequency of individuals reporting discomfort, itching, relaxation, pain relief, and improved mobility was also compared.

Student's paired samples t-test was used to compare the intensity of pain on palpation and mandibular movements in each group before and after the application of the athletic tape. A simple logistic regression was performed to compare jaw movements and pain intensity between groups. The Fisher Exact test was used to compare the sensations reported between the groups. The significance level in all analyses was set at 5%. The program used in the analyses was IBM SPSS Statistics, version 24.

## RESULTS

**Figure 2:**
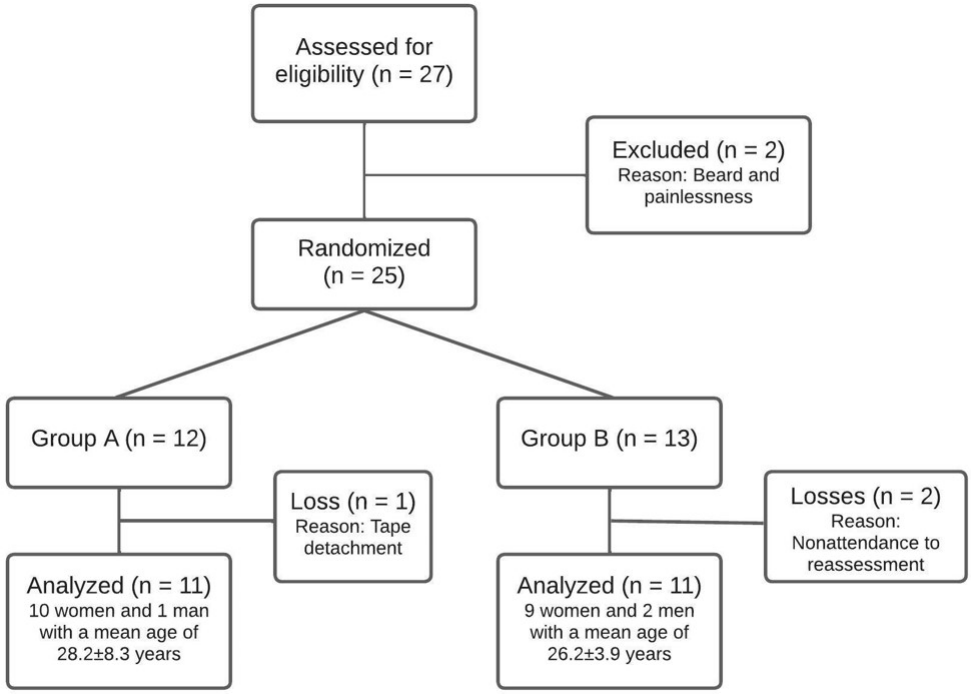
Flowchart of the study

Figure 2 presents the flowchart of the distribution of research participants. Two of the 27 individuals in the initial sample were excluded due to being painless and having a beard in the masseter region. The remaining 25 participants were randomly allocated. However, one participant in group A was excluded because of tape detachment, and two participants in group B were excluded for not attending the reassessment.

Based on the DC/TMD classification^(20)^, group A had seven individuals with myalgia, four with myalgia and arthralgia, three with disc displacement with reduction, and one with disc displacement with reduction and intermittent locking. Group B had eight individuals with myalgia, four with myalgia and arthralgia, two with disc displacement with reduction, and one with disc displacement without reduction, without limitation of opening.

Table 1 presents the analysis of participants' responses to the pain screening. Most individuals in each group reported feeling pain in the previous 30 days. In both groups, most participants reported jaw pain or stiffness upon waking. Also, most participants in both groups perceived no change in the intensity of pain when masticating hard or consistent foods, opening the mouth, moving the jaw forward or sideways, or performing other activities. However, most participants in group B reported a change in pain during deleterious oral habits.

**Table 1:** Descriptive analysis of aspects related to pain screening in the previous 30 days in groups A and B

		Group A (n = 11)		Group B (n = 11)	
		n	%	n	%
In the last 30 days, how long did any pain last in your jaw or temple area on either side?	No pain	5	45.45	4	36.36
	Pain comes and goes	4	36.36	6	54.55
	Pain is always present	2	18.18	1	9.09
In the last 30 days, have you had pain or stiffness in your jaw on awakening?	No	5	45.45	2	18.18
	Yes	6	54.55	9	81.82
In the last 30 days, did the following activities change any pain (that is, make it better or make it worse) in your jaw or temple area on either side?	A. Chewing hard or tough food				
	No	10	90.91	6	54.55
	Yes	1	9.09	5	45.45
	B. Opening your mouth or moving your jaw forward or to the side				
	No	9	81.82	6	54.55
	Yes	2	18.18	5	45.45
	C. Jaw habits such as holding teeth together, clenching, grinding, or chewing gum				
	No	6	54.55	3	27.27
	Yes	5	45.45	8	72.73
	D. Other jaw activities such as talking, kissing, or yawning				
	No	11	100.00	6	54.55
	Yes	0	0.00	5	45.45

**Caption:** n = number of participants; % = relative frequency of participants

Table 2 compares the mean pain intensity reported by participants in group A and group B before and after intervention with the tape. There was a significant difference in group A in the intensity of pain on the right TMJ and at the origin of the left masseter – the pain intensity in both regions was greater before using the tape. In group B, a significant difference was found in the sensation of pain in the anterior temporal muscle on the left side – the pain intensity was greater before the intervention.

**Table 2:** Comparison of pain intensity before and after applying the athletic tape

Points of pain measurement	Pain intensity									
	Group A (n = 11)					Group B (n = 11)				
	Before		After		p-value^1^	Before		After		p-value^1^
	Mean	Standard deviation	Mean	Standard deviation		Mean	Standard deviation	Mean	Standard deviation	
R – Posterior temporal	0.09	0.30	0.64	1.21	0.167	1.64	3.04	1.09	2.21	0.192
L – Posterior temporal	0.91	1.30	0.55	1.21	0.531	0.91	1.64	1.00	1.79	0.863
R – Middle temporal	1.27	2.15	1.18	1.72	0.831	1.91	2.59	1.18	1.60	0.104
L – Middle temporal	1.18	1.78	1.00	1.84	0.774	1.73	2.87	1.00	1.55	0.167
R – Anterior temporal	1.82	3.31	1.18	1.99	0.370	2.18	2.27	1.27	1.85	0.085
L – Anterior temporal	1.82	2.40	0.64	1.12	0.109	1.64	2.58	1.09	2.07	0.025*
R – TMJ	1.64	2.50	0.45	1.04	0.040*	1.36	1.75	1.00	1.95	0.553
L – TMJ	0.45	1.04	0.36	0.81	0.796	1.82	2.27	1.09	1.45	0.356
R – Open TMJ	1.73	2.94	1.55	2.84	0.881	1.27	2.28	1.00	2.14	0.192
L – Open TMJ	1.00	1.55	1.00	1.84	0.999	2.18	2.89	1.00	1.55	0.168
R – Origin masseter	2.45	3.21	2.18	2.75	0.661	2.73	2.57	1.82	1.83	0.157
L – Origin masseter	2.55	2.91	1.00	1.34	**0.036** *	2.18	2.64	1.55	1.75	0.361
R – Middle masseter	2.64	2.91	3.45	2.94	0.314	4.09	3.75	3.18	3.03	0.148
L – Middle masseter	3.00	2.68	2.82	3.19	0.859	4.27	3.32	3.45	3.42	0.268
R – Insertion masseter	2.82	2.56	2.45	2.98	0.623	4.27	3.00	2.73	2.97	0.068
L – Insertion masseter	3.64	3.17	3.36	3.11	0.493	4.18	2.99	3.36	3.17	0.213

1 Student’s paired samples t-test

* p < 0.050

**Caption:** TMJ = temporomandibular joint; R = right; L = left

The comparison of the range of mandibular movements before and after intervention is shown in Table 3. There was no significant difference between the mean range of mandibular movements between these moments in either group.

**Table 3:** Comparison of the range of mandibular movement before and after applying the athletic tape

Mandibular movements	Range of mandibular movements									
	Group A (n = 11)					Group B (n = 11)				
	Before (mm)		After (mm)		p-value^1^	Before (mm)		After (mm)		p-value^1^
	Mean	Standard deviation	Mean	Standard deviation		Mean	Standard deviation	Mean	Standard deviation	
Painless opening	47.13	6.89	46.88	7.55	0.811	46.14	9.06	46.32	11.37	0.953
Unassisted mouth opening	54.15	5.38	53.49	5.14	0.151	54.04	7.82	50.96	12.80	0.286
Assisted mouth opening	56.10	6.34	56.48	6.09	0.403	55.69	8.46	52.70	12.94	0.290
Lateralization to the right	8.70	2.09	9.17	1.79	0.307	8.26	2.80	8.14	3.72	0.795
Lateralization to the left	9.14	2.47	9.13	2.02	0.981	7.75	3.41	8.47	2.20	0.491
Protrusion	7.69	1.29	7.86	0.91	0.668	8.77	2.19	8.71	1.76	0.800

1 Student’s paired samples t-test

No significant difference was found in the reduction of pain intensity or the range of mandibular movements between the groups (Table 4).

**Table 4:** Comparison of the reduction of pain intensity and the difference in the range of mandibular movements between the groups after applying the athletic tape

Dependent variables	Independent variable	Group B	Standard error	p-value*	95% CI for Group B	
					Lower limit	Upper limit
R – Posterior temporal	Group A	-0.455	0.760	0.556	-2.039	1.130
L – Posterior temporal	Group A	-0.455	0.652	0.494	-1.814	0.905
R – Middle temporal	Group A	0.034	0.709	0.999	-1.479	1.589
L – Middle temporal	Group A	0.012	0.726	0.999	-1.515	1.785
R – Anterior temporal	Group A	-0.091	0.819	0.913	-1.800	1.618
L – Anterior temporal	Group A	-0.455	0.710	0.529	-1.936	1.027
R – TMJ	Group A	-0.545	0.666	0.422	-1.934	0.843
L – TMJ	Group A	-0.727	0.500	0.161	-1.769	0.315
R – Open TMJ	Group A	0.545	1.073	0.617	-1.694	2.784
L – Open TMJ	Group A	0.100	0.726	0.999	-1.515	1.578
R – Origin masseter	Group A	0.364	0.997	0.719	-1.715	2.443
L – Origin masseter	Group A	-0.545	0.666	0.422	-1.934	0.843
R – Middle masseter	Group A	0.273	1.273	0.833	-2.383	2.929
L – Middle masseter	Group A	-0.636	1.409	0.656	-3.575	2.303
R – Insertion masseter	Group A	-0.273	1.268	0.832	-2.918	2.373
L – Insertion masseter	Group A	0.187	1.339	0.999	-2.792	2.988
Painless opening	Group A	0.564	4.116	0.892	-8.021	9.149
Unassisted opening	Group A	2.527	4.160	0.550	-6.150	11.204
Assisted opening	Group A	3.782	4.311	0.391	-5.211	12.775
Lateralization to the right	Group A	1.021	1.245	0.422	-1.577	3.619
Lateralization to the left	Group A	0.655	0.900	0.475	-1.223	2.534
Protrusion	Group A	-0.845	0.597	0.172	-2.090	0.399

Note: Only data after applying the athletic tape

(*) Simple linear regression

**Caption:** B - Coefficient; B is the reference group; significant if p ≤ 0.050; TMJ = temporomandibular joint; R = right; L = left; CI= confidence interval; Group A had n = 11; Group B had n = 11

Table 5 presents the association of sensations reported after using the tape between the two groups. There was no significant difference in the sensations reported between them. Therefore, discomfort, itching, a feeling of relaxation, pain, and increased mobility do not depend on whether the tape is applied without stretch or with 40% stretch.

**Table 5:** Comparison of sensations reported after using the athletic tape between the two groups

Reported sensations		Group A (n = 11)		Group B (n = 11)		p-value^1^
		n	%	n	%	
Sensation of discomfort	No	7	63.64	7	63.64	0.999
	Yes	4	36.36	4	36.36	
Sensation of itching	No	9	81.82	10	90.91	0.999
	Yes	2	18.18	1	9.09	
Sensation of relaxation	No	6	54.55	7	63.64	0.999
	Yes	5	45.45	4	36.36	
Sensation of eased pain	No	9	81.82	10	90.91	0.999
	Yes	2	18.18	1	9.09	
Sensation of improved mobility	No	9	81.82	9	81.82	0.999
	Yes	2	18.18	2	18.18	

1 Fisher’s exact test

**Caption:** n = number of participants; % = relative frequency of participants

## DISCUSSION

The hypothesis examined in this study assumed that using the tape over the masseter would ease pain and increase mandibular mobility, with the greatest effects by applying the tape with stretching. The results do not confirm this hypothesis since mobility did not improve, and the pain eased in isolated points. Furthermore, there was no difference between the groups.

No articles were found in the literature that evaluated the effects of using the tape over the masseter alone for 24 hours. A study investigated the use of athletic tape with 40% stretch for 5 weeks in patients with sleep bruxism and showed that, after 1 week, participants felt decreased pain in their masticatory muscles^(18)^. Another study^(19)^ suggests the benefits of using athletic tapes for a week, with light stretching (0% to 15%) associated with guidance and jaw exercises in patients with TMD, reducing their pain on palpation in the masseter and temporal muscles and the joint. Lietz-Kijak^(17)^ and her team found a reduction in the intensity of orofacial pain after 5 days of using the tape on the masseter without stretching. The short time of tape use in this research – along with using it in isolation, without other interventions, such as counseling – is believed to be one of the reasons for the little improvement in painful symptoms.

Lemos^(13)^ lists the benefits of using athletic tape in four functions: dermal, muscular, joint, and lymphatic. The effects on dermal function occur in response to sensory stimulation caused by contact of the tape with the skin, activating cutaneous mechanoreceptors and, consequently, peripheral nerves through tension, pressure, decompression, and elevation of the skin^(13)^. Stimulation of mechanoreceptors located in the skin causes the activation of afferent neurons, whose bodies are in the dorsal ganglia of the spinal cord. The mechanical stimulus generated by the tape competes in the afferent pathways with the pain stimulus, inhibiting it^(12,13)^. Moreover, as the tape elevates the skin, it decompresses nociceptors by reducing local edema^(13)^. In addition to relieving pain and discomfort locally and in underlying tissues, the tape promotes greater movement proprioception and joint alignment. When applied with a fixed point at the muscle insertion, the tape also causes a recession of the muscle action, relaxing hyperfunctional muscles^(13)^.

Since the tape acts mainly at the skin level, a greater reduction in pain intensity was expected in group A, with greater stretch and, therefore, applying sensory stimulus at greater intensity. In this regard, the present research results align with the hypothesis, as the pain in group A reduced in more measurement points – which may have been due to the greater sensory stimulus applied, compared to group B. However, using the tape for just 24 hours may not have been enough to relieve pain in more areas. Another possible explanation for not having found a significant difference in pain reduction was the participants’ low level of pain in the initial assessment, whose mean was lower than 4.5 in the points evaluated, thus giving little room for change in the level of pain in the final analysis.

The use of athletic tape in this study was unable to change mouth opening or provide greater jaw lateralization or protrusion. This result was supposedly influenced by the absence of jaw mobility exercises or other therapeutic approaches in the intervention, such as myofunctional therapy, and the 24-hour usage time^(19)^. The study by Keskinruzgar and collaborators^(18)^ found that the application of athletic tape increased mouth opening after 5 weeks in patients with bruxism. However, in the authors’ measurement in the first week of tape use, the results had not yet indicated a significant improvement. The study by Benlidayi and team^(19)^ provided treatment with the tape associated with physical therapy exercises for 6 weeks in 28 patients with TMD and verified an improvement in the functional limitation of maximum mouth opening and an increase in right lateral movements in the study group after 6 weeks, which cannot be ascribed to the tape use alone. In the study by Ozmen and collaborators^(23)^, the authors found an increase in mandibular mobility in the group that used tapes. However, it was likewise not applied alone, but in combination with prescribed anti-inflammatory and muscle-relaxing medications, occlusal splint, and exercises.

The present study found no difference between groups A and B for either pain or range of motion after using the tape for one day. This result agrees with Emérito and collaborators^(12)^ in their systematic review, which showed that the use of athletic tape over the masseter muscle reduces pain after 1 week of treatment. It is speculated that, in this sense, longer treatments could promote differences between the groups. As there are no studies investigating such outcomes after 24 hours of tape use, this investigation was considered important, and it is suggested that future studies use different stretches and durations of tape use.

Tape use with 40% stretch in the present study was based on the one by Keskinruzgar and collaborators^(18)^, which had positive results for pain and mouth opening in individuals with bruxism after using the tape with this tension. The tape was applied without stretching in the present study to form a control group and verify the effect of 40% stretch. Despite the lack of significant difference between the groups, it is noteworthy that pain relief was perceived at more points in the group that used the tensioned tape.

According to the results, the sensations reported after using the tape were not different between the groups. Therefore, experiencing sensations of relaxation, discomfort, itching, pain, or increased mobility is independent of the tape stretch applied for 24 hours. Most individuals in both groups reported not noticing relaxation or improvement in pain. However, body perception is subjective and may depend on other factors, such as the subject's personal history^(16)^, as well as the sensation of reduced pain, which may have gone unnoticed by some individuals^(24)^.

The limitations of this study were the small and heterogeneous sample regarding the TMD diagnosis, the loss of participants during collection, the little time of tape use, and the participants’ mild pain, whose values were in general less than 4. Therefore, the results of this research should be approached with caution and not generalized. As a strength, this study is unprecedented, being the first in the scientific world to investigate the interference of athletic tape on pain and mandibular movements with different stretches in randomized double-blind research.

## CONCLUSION

The use of athletic tape for 24 hours on the masseter muscle with a 40% stretch relieved the study population’s sensation of pain only in the origin of the left masseter muscle and the right TMJ. Using it without stretching reduced pain in the left anterior temporal muscle. There was no difference in the range of mandibular movements after using the tape with or without stretching for 24 hours.

## References

[1] Conti PCR, Gonçalves DAG (2022). International Classification of Orofacial Pain – ICOP – Brazilian Portuguese version. Headache Med..

[2] American Association of Dental Research ( 2015). Temporomandibular Disorders (TMD) 2015.

[3] Sassi FC, Silva AP, Santos RKS, Andrade CRF (2018). Tratamento para disfunções temporomandibulares: uma revisão sistemática. Audiol Commun Res.

[4] Kalladka M, Young A, Khan J (2021). Myofascial pain in temporomandibular disorders: updates on etiopathogenesis and management. J Bodyw Mov Ther.

[5] Bueno CH, Pereira DD, Pattussi MP, Grossi PK, Grossi ML (2018). Gender differences in temporomandibular disorders in adult populational studies: a systematic review and meta‐analysis. J Oral Rehabil.

[6] Brandão RAFS, Mendes CMC, Lopes TDS, Brandão RA, Sena EP (2021). Neurophysiological aspects of isotonic exercises in temporomandibular joint dysfunction syndrome. CoDAS.

[7] Gałczyńska-Rusin M, Pobudek-Radzikowska M, Prylińska-Czyżewska A, Maciejewska-Szaniec Z, Gawriołek K, Strużycka I (2021). Comparison of the fffects of myotherapy in patients with myofascial pain with and without self-reported sleep bruxism using The Research Diagnostic Criteria for Temporomandibular Disorders (RDC/TMD) Axis I Questionnaire. Med Sci Monit.

[8] Olchowy A, Seweryn P, Olchowy C, Wieckiewicz M (2022). Assessment of the masseter stiffness in patients during conservative therapy for masticatory muscle disorders with shear wave elastography. BMC Musculoskelet Disord.

[9] He F, Ma Y, Yu B, Ji R, Lu J, Chen W (2020). Preliminary application of Kinesio taping in rehabilitation treatment of temporomandibular disorders. Iran Red Crescent Med J.

[10] Hernandes NCJ, Ribeiro LL, Gomes CF, Silva AP, Dias VF (2017). Speech therapy in temporomandibular dysfunction in two cases: comparative analysis of the effect of traditional therapy and the use of the therapeutic bandage associated. Distúrb Comun.

[11] Cheshmi B, Keyhan SO, Rayegani SM, Kim SG, Ozunlu Pekyavas N, Ramezanzade S (2021). A literature review of applications of Kinesio Taping® in the craniomaxillofacial region. Cranio.

[12] Emérito TM, Silva JAS, Furlan RMMM (2022). Use of kinesiology tape for pain relief in the treatment of temporomandibular disorders: a systematic review with meta-analysis. Audiol Commun Res.

[13] Lemos T, Kase K, Dias E (2009). Kinesio Taping® Introdução ao Método e Aplicações Musculares..

[14] Emérito TM (2020). Elastic bandage as a therapeutic resource in orofacial motricity: a bibliographic study. Pubsaúde..

[15] Tran L, Makram AM, Makram OM, Elfaituri MK, Morsy S, Ghozy S (2023). Efficacy of kinesio taping compared to other treatment modalities in musculoskeletal disorders: a systematic review and meta-analysis. Res Sports Med.

[16] Silva AP, Carvalho ARR, Sassi FC, Silva MAA (2019). The taping method effects on the trapezius muscle in healthy adults. CoDAS.

[17] Lietz-Kijak D, Kopacz L, Ardan R, Grzegocka M, Kijak E (2018). Assessment of the short-term effectiveness of kinesiotaping and trigger points release used in functional disorders of the masticatory muscles. Pain Res Manag.

[18] Keskinruzgar A, Kucuk AO, Yavuz GY, Koparal M, Caliskan ZG, Utkun M (2019). Comparison of kinesio taping and occlusal splint in the management of myofascial pain in patients with sleep bruxism. J Back Musculoskelet Rehabil.

[19] Benlidayi IC, Salimov F, Kurkcu M, Guzel R (2016). Kinesio Taping for temporomandibular disorders: single-blind, randomized, controlled trial of effectiveness. J Back Musculoskeletal Rehabil.

[20] Schulz KF, Altman DG, Moher D, CONSORT Group (2010). CONSORT 2010 Statement: updated guidelines for reporting parallel group randomised trials.

[21] Ohrbach R (2016). Diagnostic criteria for temporomandibular disorders: assessment instruments. Version 15.

[22] Cortese SG, Oliver LM, Biondi AM (2007). Determination of range of mandibular movements in children without temporomandibular disorders. Cranio.

[23] Özmen EE, Durmuş E, Ünüvar BS, Kalayci A (2022). Mid-and long-term effect of Kinesio Taping on temporomandibular joint dysfunction: A Randomised-Controlled Trial. Acıbadem Üniversitesi Sağlık Bilimleri Dergisi..

[24] Smith SM, Dworkin RH, Turk DC, McDermott MP, Eccleston C, Farrar JT (2020). Interpretation of chronic pain clinical trial outcomes: IMMPACT recommended considerations. Pain.

