# Supplementary material for: Effects of athletic tape on orofacial pain and jaw movements after 24 hours of use: a randomized clinical trial
Source: Codas. 2024 May 27;36(3):e20230066. doi: 10.1590/2317-1782/20242023066en (PMC11166035; doi:10.1590/2317-1782/20242023066en)
Supplement: Translation

# Translation: Efeitos da bandagem elástica na dor orofacial e nos movimentos mandibulares após 24 horas de uso: ensaio clínico randomizado

**DOI:** 10.1590/2317-1782/20242023066pt

**Authors:** Marília dos Santos Faria, Gabriela Carolina Silva Teodoro, Júlia Ana Soares Silva, Tatyana Meneses Emérito, Andréa Rodrigues Motta, Mariana Souza Amaral, Renata Maria Moreira Moraes Furlan

**Affiliations:** 1 Curso de graduação em Fonoaudiologia, Faculdade de Medicina, Universidade Federal de Minas Gerais – UFMG - Belo Horizonte (MG), Brasil.; 2 Curso de graduação em Fonoaudiologia, Centro Universitário Uninovafapi – UNINOVAFAPI - Teresina (PI) - Brasil.; 3 Departamento de Fonoaudiologia, Faculdade de Medicina, Universidade Federal de Minas Gerais – UFMG - Belo Horizonte (MG), Brasil.; 4 Programa de Pós-graduação em Ciências Fonoaudiológicas, Universidade Federal de Minas Gerais – UFMG - Belo Horizonte (MG), Brasil.

**Keywords:** Bandagem Elástica, Dor, Dor Orofacial, Transtornos da Articulação Temporomandibular, Músculos da Mastigação

## Abstract

**Objetivo:**

Analisar a sensação de dor e amplitude dos movimentos mandibulares de indivíduos adultos com disfunção temporomandibular, antes e após aplicação da bandagem elástica por 24 horas.

**Método:**

Trata-se de um ensaio clínico randomizado duplo-cego, do qual participaram 22 sujeitos adultos com disfunção temporomandibular, alocados aleatoriamente em dois grupos, sendo grupo A composto por 10 mulheres e um homem (média de idade de 28,2±8,3 anos) e grupo B por nove mulheres e dois homens (média de idade de 26,2±3,9 anos). Todos os participantes foram submetidos à aplicação do Diagnostic Criteria for Temporomandibular Disorders (DC/TMD). Foi realizada a avaliação do limiar da dor, com uso de um algômetro, para aplicação da pressão no masseter e temporal e medição dos movimentos mandibulares, com paquímetro. O grupo A foi submetido à aplicação da bandagem sobre o músculo masseter com estiramento de 40% e o grupo B sem estiramento. A colagem da bandagem foi realizada, com corte em “I”, com ponto fixo sobre a inserção e ponto móvel sobre a origem do músculo masseter. Os participantes permaneceram com a bandagem por 24 horas e foram reavaliados.

**Resultados:**

Houve alívio da dor no grupo A na articulação temporomandibular à direita e na origem do masseter à esquerda. O grupo B apresentou redução da dor em região de temporal anterior à esquerda. Não foram encontradas diferenças nos movimentos mandibulares após intervenção, bem como não houve diferença na comparação entre os grupos.

**Conclusão:**

O uso da bandagem sobre o masseter, por 24 horas, com estiramento, produziu alívio da dor na origem do masseter direito e na região da articulação temporomandibular direita e, sem estiramento, no temporal anterior esquerdo. Não houve diferença na amplitude de movimentos mandibulares.

## INTRODUÇÃO

A dor orofacial é conhecida na literatura como um termo amplo que abrange sensações dolorosas na face e boca^(1)^. É possível ser atribuída à disfunção temporomandibular (DTM), a desordens de estruturas dentoalveolares e anatomicamente relacionadas, à lesão ou doença dos nervos cranianos, a dores orofaciais com manifestações semelhantes a cefaleias primárias e à dor orofacial idiopática^(1)^.

A DTM é uma alteração que compreende quadros musculoesqueléticos e neuromusculares que abrangem a disfunção da articulação temporomandibular (ATM) e todos os músculos e tecidos relacionados, sendo que as alterações nas funções de mastigação, deglutição e fala, bem como a dor aguda ou persistente são sintomas frequentes desta disfunção^(2)^. Além disso, a DTM está diretamente relacionada aos movimentos e atividades funcionais dos músculos da mastigação, e comumente é evidenciada pela redução da amplitude dos movimentos mandibulares^(3)^. Sua etiologia é multifatorial, contendo como elementos hábitos orais, traumas, má-oclusão, alterações de postura, estresse e ansiedade^(3,4)^. Estudos evidenciam a prevalência maior em mulheres adultas, duplicando a chance do desenvolvimento de DTM no sexo feminino; outros fatores como idade, etnia e fatores psicossociais também devem ser considerados^(5)^.

Diversas técnicas têm sido utilizadas durante o processo terapêutico para disfunção temporomandibular, tais como: exercícios ativos de relaxamento^(6)^, alongamento^(7)^, massagem^(8)^, estabilização oclusal^(8)^, educação em saúde e aplicação de ondas curtas^(9)^, entre outros. Recentemente tem sido indicada a aplicação da bandagem elástica^(10-12)^ que tem demonstrado excelentes resultados no alívio da dor, relaxamento muscular, fortalecimento dos músculos debilitados, melhora da circulação sanguínea e linfática^(12)^.

A bandagem elástica surgiu em 1970 no Japão, tendo sido criada por Kenzo Kase, um quiropraxista japonês com o intuito de auxiliar atletas no tratamento de lesões e na reabilitação das disfunções musculoesqueléticas^(13-15)^. Desde então, vem sendo utilizada por diversos profissionais para fins de reabilitação ou estimulação muscular, como terapeutas ocupacionais, fisioterapeutas e fonoaudiólogos.

A composição da bandagem elástica baseia-se em algodão retorcido envolvendo micro fios de elastano, sem adição medicamentosa, que adere na pele humana e pode ser estirada permanecendo por tempo prolongado^(16)^. Pode ser aplicada com diferentes estiramentos (0% a 100%) e possui objetivos dérmicos, musculares, articulares e linfáticos, dentre eles: aumento da sensibilidade da pele, excitação ou inibição muscular, aumento da circulação sanguínea e linfática, e redução da dor^(16)^. Estudos recentes evidenciaram que o uso da bandagem elástica proporciona efeitos analgésicos^(17)^, incluindo redução da dor muscular^(18)^, além de aumento da abertura de boca^(18)^ e, associado a exercícios dos músculos mastigatórios, aumento da mobilidade da ATM^(19)^.

Emérito^(14)^ realizou um estudo bibliográfico acerca do uso da bandagem elástica como recurso terapêutico na Motricidade Orofacial e concluiu que o método é eficaz no tratamento das alterações e disfunções orofaciais, tornando-se uma alternativa para os profissionais envolvidos neste tratamento.

Uma revisão sistemática da literatura com metanálise^(12)^ verificou o efeito da aplicação da bandagem elástica sobre músculos mastigatórios no alívio da dor, em comparação com outras intervenções, em indivíduos com disfunções temporomandibulares. A conclusão encontrada refere que o uso da bandagem elástica denota resultados significativos para diminuição da dor na primeira semana.

O número de estudos sobre o uso da bandagem elástica tem se ampliado, entretanto, a literatura, no que se refere ao uso deste método para dor orofacial e disfunções temporomandibulares, ainda é reduzida e os efeitos relacionados ao maior ou menor estiramento da bandagem não são conhecidos. Além disso, não foram encontrados estudos que avaliassem os efeitos imediatos da bandagem, mas sim efeitos em tempo maior, como cinco dias consecutivos^(17)^, 35 dias^(18)^ e seis semanas^(19)^, sendo importante verificar se aplicações isoladas por curto período já produzem algum efeito.

Desta forma, o objetivo do presente estudo foi analisar a sensação de dor e a amplitude dos movimentos mandibulares de indivíduos adultos com disfunção temporomandibular, antes e após a aplicação da bandagem elástica por 24 horas. Tem-se como hipóteses que o uso da bandagem sobre o músculo masseter proporciona, por meio do efeito sensorial percebido, uma postura mandibular livre de apertamento dentário e, consequentemente, alívio da sensação de dor orofacial nos músculos elevadores da mandíbula masseter e temporal, bem como aumento na abertura da boca, na lateralização e protrusão de mandíbula; e que a aplicação da bandagem com estiramento produz maiores efeitos do que a aplicação sem estiramento.

## MÉTODO

Trata-se de um ensaio clínico randomizado duplo-cego, realizado com 22 estudantes da Faculdade de Medicina da Universidade Federal de Minas Gerais (UFMG), com DTM, sendo a amostra de conveniência. A coleta foi realizada de abril a julho de 2022 no Observatório de Saúde Funcional em Fonoaudiologia da Faculdade de Medicina da UFMG. O estudo foi realizado conforme diretrizes do *CONSORT Reporting Guidelines* ^(20)^, tendo sido aprovado pelo Comitê de Ética em Pesquisa sob parecer de número 4.329.360 (CAAE 36777220.7.0000.5149) e registrado na plataforma REBEC sob o número RBR-46cmrsb. Todos os participantes do estudo assinaram o Termo de Consentimento Livre e Esclarecido, concordando com os termos de pesquisa.

Para a seleção dos participantes foi realizada a divulgação da pesquisa no *Campus* da UFMG e por plataformas virtuais. Foram incluídos indivíduos acima de 18 anos, de ambos os sexos, com DTM. Foram excluídos do estudo indivíduos com ausência de dor orofacial à palpação, que apresentaram feridas na área da aplicação da bandagem elástica, indivíduos com histórico alérgico ao uso de bandagem, com má-formação facial, alteração neurológica ou doença neurodegenerativa, em uso de analgésicos, relaxantes musculares ou anti-inflamatórios e aqueles que não permaneceram com a bandagem pelo tempo predeterminado.

Todos os participantes preencheram uma ficha que continha questões relacionadas a queixas orofaciais, mastigação, dores orofaciais, histórico alérgico, uso de medicamentos e diagnóstico neurológico para avaliação dos critérios de elegibilidade. Durante a primeira sessão, a pesquisadora 1 realizou os seguintes procedimentos: anamnese, aplicação do protocolo *Diagnostic Criteria for Temporomandibular Disorders (DC/TMD)* ^(21)^, registrou a avaliação da intensidade da dor à palpação e a medição da abertura de boca, protrusão e lateralização mandibular direita e esquerda.

Para a avaliação da dor, foi utilizado o algômetro (Marca Kilter, modelo FM-207), instrumento que possibilita a aplicação de pressões conhecidas em diferentes pontos da face. Aplicou-se 0,5 Kg de pressão na ATM e 1 Kg nos músculos masseteres (origem, região média e inserção) e temporais (médio, anterior e posterior) e, em seguida, o participante foi solicitado a informar seu nível de dor em uma escala graduada de 0 a 10, sendo que 0 indicava nenhuma dor e 10 a máxima dor possível^(21)^.

Posteriormente, a avaliadora 1 forneceu modelo visual dos movimentos mandibulares de abertura de boca, lateralização e protrusão mandibular e, em seguida, solicitou que o voluntário repetisse os movimentos para medição com uso do paquímetro (Digimess®, São Paulo). As medidas registradas foram: trespasse vertical, distância interincisal máxima ativa e lateralidade mandibular direita e esquerda, conforme descrito no DC^(21)^ .

Para a avaliação da abertura de boca, foram realizadas três medições: abertura sem dor, abertura máxima não assistida e abertura assistida. Em todas as medições, a abertura máxima de boca foi considerada como sendo a distância da borda incisal dos dentes incisivos superiores aos inferiores. Primeiramente o participante foi solicitado a realizar a maior abertura sem dor, em seguida a maior abertura independente da dor e, por último, a maior abertura com auxílio manual da pesquisadora exercendo uma força na mandíbula no sentido da abertura^(21)^. A medida do trespasse vertical foi somada à medida da abertura de boca.

Para avaliar a lateralidade mandibular, foi solicitado que o participante deslocasse ao máximo a mandíbula para o lado direito, medindo-se a distância horizontal entre a linha média dentária dos incisivos inferiores e a linha média dentária dos incisivos superiores^(22)^. O mesmo procedimento foi realizado para o lado oposto, considerando a medida do deslocamento da linha média. Quando as linhas médias superior e inferior não eram coincidentes (distância maior que 1 mm), anotou-se o valor e a direção da discrepância. A linha média superior foi considerada como referência e o valor da discrepância foi subtraído na lateralização ipsilateral e somado na lateralização contralateral.

Para mensurar a protrusão mandibular, a partir dos dentes em oclusão, foi medida a distância horizontal entre a região medial da face vestibular dos incisivos inferiores e a borda incisal dos superiores. Em seguida, foi solicitado que os participantes protruíssem a mandíbula e, a medida foi novamente aferida, a partir da distância entre a face vestibular dos incisivos centrais inferiores e a borda incisal dos incisivos superiores, que foram somadas para se obter a distância percorrida pela mandíbula na protrusão.

Foram realizadas três medições em cada uma dessas situações acima mencionadas, sendo desconsiderada a primeira, e considerada apenas a medida de maior valor encontrado.

Para a aplicação da bandagem elástica, os participantes foram alocados pela pesquisadora 2 nos grupos A e B de forma randomizada por sorteio. O grupo A foi composto por 11 indivíduos (10 mulheres e um homem) com média de idade de 28,2±8,3 anos e o grupo B foi composto por 11 indivíduos (nove mulheres e dois homens) com média de idade de 26,2±3,9 anos.

O grupo A foi submetido à aplicação da bandagem sobre o músculo masseter com estiramento de 40%^(18)^ e o grupo B foi submetido à aplicação da bandagem sobre o músculo masseter sem estiramento, configurando o grupo controle. A pesquisadora 2 aplicou a bandagem elástica da marca Kinésio Tmax (aprovada pela Agência Nacional de Vigilância Sanitária, sob o registro 10410130023) sobre o músculo masseter usando o corte em “I”, com ponto de ancoramento inicial no ângulo da mandíbula^(13)^. O tamanho da fita, na largura, foi de 2,5 cm, enquanto, no eixo longitudinal, foi definido de acordo com as características anatômicas individuais de cada participante.

Quando aplicada sem estiramento o corte da bandagem tinha, como comprimento, a medida do arco zigomático até o ângulo da mandíbula de cada indivíduo. Para a aplicação com estiramento de 40%, o comprimento da bandagem precisava ser menor e calculado especificamente para cada indivíduo. A partir da diferença entre o comprimento do corte de bandagem estirado a 100% e o comprimento desse corte sem o estiramento, obteve-se o tamanho do aumento de comprimento no estiramento de 100% (delta B). Em seguida, por regra de três, calculou-se o comprimento original da fita para que o estiramento fosse apenas 40% de delta B.

Para aplicação da bandagem, foi realizada a limpeza da região da pele sobre o músculo masseter, com gaze embebida em álcool 70%. Em seguida, a pesquisadora 2 realizou a medição do músculo masseter desde a sua origem (arco zigomático) até sua inserção (ângulo da mandíbula), com escala de medidas que se encontra no verso da bandagem. Após cortar a bandagem, foi realizado o arredondamento dos cantos da bandagem para melhor fixação dessa e a marcação da zona terapêutica e âncoras por meio de uma dobradura na bandagem. A colagem foi realizada do ponto fixo para o ponto móvel (Figura 1). Para tanto, a ancoragem inicial (ponto fixo) foi realizada no ângulo da mandíbula, em seguida foi solicitado que o participante abrisse a boca (abertura máxima), então foi realizado o estiramento da bandagem^(18)^ no grupo A para realização da colagem na zona terapêutica. O Grupo B foi submetido ao mesmo procedimento, porém a bandagem não foi estirada. A bandagem foi aplicada bilateralmente em todos os participantes e cada participante permaneceu com a bandagem por 24 horas, sem alteração da rotina diária.

**Figura 1:** Bandagem elástica aplicada sobre a região do músculo masseter

A pesquisadora 1 (responsável pela avaliação dos participantes) não se encontrava presente na sala de coleta no momento da aplicação da bandagem, estando cega quanto a qual grupo o participante pertencia. Da mesma forma, a pesquisadora 2 (responsável pela alocação dos participantes nos grupos e pela aplicação da bandagem) não estava presente no momento das avaliações dos participantes. O participante também estava cego quanto ao grupo ao qual foi alocado.

Após 24 horas, os voluntários retornaram para a segunda sessão, na qual a pesquisadora 1 retirou a bandagem elástica, aferiu o nível de dor usando o algômetro e mediu a abertura da boca, lateralização e protrusão mandibular. Assim, em ambos os grupos, a pesquisadora que realizou a avaliação foi sempre diferente daquela que aplicou a bandagem, sendo que a primeira não sabia a qual grupo o indivíduo pertencia.

Após a reavaliação, os participantes responderam um questionário com o intuito de verificar a percepção quanto ao uso da bandagem. O questionário foi baseado no estudo de Silva et al.^(16)^, sendo composto de cinco questões fechadas, com opções de resposta “sim” ou “não”, para verificação das possíveis sensações causadas pelo seu uso: incômodo, coceira, relaxamento, alívio da dor e melhora da mobilidade.

As variáveis resposta do estudo foram: intensidade da dor orofacial e amplitude dos movimentos mandibulares. Estas variáveis foram comparadas nos momentos de avaliação e reavaliação para cada grupo. As variáveis explicativas foram o momento (pré vs pós intervenção) e o estiramento da bandagem (grupo A vs grupo B). Os dados foram registrados em uma planilha do Microsoft Excel e, posteriormente, analisados por meio de medidas de tendência central e dispersão para as variáveis contínuas e por meio de distribuição de frequência para as variáveis categóricas. Também foi comparada a frequência de indivíduos que relataram sensação de incômodo, coceira, relaxamento, alívio de dor e melhora da mobilidade.

Utilizou-se o teste *t* de *Student* para amostras pareadas nas comparações da intensidade da dor à palpação e dos movimentos mandibulares em cada grupo entre os momentos antes e após a aplicação da bandagem elástica. Foi realizada uma regressão logística simples para comparar os movimentos mandibulares e a intensidade da dor entre os grupos. O teste Exato de Fisher foi utilizado na comparação das sensações reportadas entre os grupos. O nível de significância utilizado em todas as análises foi de 5%. O programa utilizado nas análises foi o IBM SPSS *Statistics version* 24.

## RESULTADOS

A Figura 2 apresenta o fluxograma da distribuição dos participantes da pesquisa. Dos 27 indivíduos da amostra inicial, dois foram excluídos por não apresentarem dor e pela presença de barba na região de masseter. Os demais 25 participantes foram alocados de forma randomizada, entretanto, um participante do grupo A foi excluído devido ao descolamento da bandagem e dois participantes do grupo B foram excluídos por não comparecerem à reavaliação.

**Figura 2:** Fluxograma do estudo

Com relação à classificação da DTM, segundo o DC/DTM^(20)^, o grupo A apresentou sete indivíduos com mialgia e quatro com mialgia e artralgia, sendo três com deslocamento do disco com redução e um com deslocamento de disco com redução com travamento intermitente. Já o grupo B apresentou oito indivíduos com mialgia e quatro com mialgia e artralgia, sendo dois com deslocamento do disco com redução e um como deslocamento do disco sem redução, sem limitação de abertura.

A Tabela 1 apresenta a análise das respostas dos participantes na triagem de dor. A maior parte dos indivíduos de cada grupo relatou presença de dor nos últimos 30 dias. Em ambos os grupos, a maioria dos participantes relatou dor ou rigidez na mandíbula ao acordar. Em ambos os grupos, para a maior parte dos participantes, não há mudança da intensidade da dor ao mastigar alimentos duros ou consistentes, ao abrir a boca ou movimentar a mandíbula para frente ou para o lado e ao realizar outras atividades. No entanto, no grupo B, a maioria dos participantes relatou mudança da dor durante hábitos orais deletérios.

**Tabela 1:** Análise descritiva dos aspectos relacionados à triagem da dor dos últimos 30 dias nos grupos A e B

		Grupo A (n=11)		Grupo B (n=11)	
		N	%	n	%
Nos últimos 30 dias, quanto tempo durou qualquer dor que você teve na mandíbula ou na região temporal em qualquer um dos lados?	Não tive dor	5	45,45	4	36,36
	Dor aparecia e desaparecia	4	36,36	6	54,55
	Dor estava sempre presente	2	18,18	1	9,09
Nos últimos 30 dias, você teve dor ou rigidez na sua mandíbula ao acordar?	Não	5	45,45	2	18,18
	Sim	6	54,55	9	81,82
Nos últimos 30 dias, as seguintes atividades mudaram qualquer dor (isto é, fizeram ela melhorar ou piorar) na sua mandíbula ou região temporal em qualquer um dos lados?	A. Mastigar alimentos duros ou consistentes				
	Não	10	90,91	6	54,55
	Sim	1	9,09	5	45,45
	B. Abrir a boca ou movimentar a mandíbula para frente ou para o lado				
	Não	9	81,82	6	54,55
	Sim	2	18,18	5	45,45
	C. Hábitos ou manias com a mandíbula (boca), como manter os dentes juntos, apertar ou ranger os dentes, ou mastigar chiclete?				
	Não	6	54,55	3	27,27
	Sim	5	45,45	8	72,73
	D. Outras atividades com a mandíbula (boca) como falar, beijar, bocejar				
	Não	11	100,00	6	54,55
	Sim	0	0,00	5	45,45

**Legenda:** n = número de participantes; % = frequência relativa de participantes

A Tabela 2 apresenta a comparação da média da intensidade de dor reportada pelos participantes do grupo A e do grupo B nos momentos pré e pós-intervenção utilizando a bandagem. Houve diferença significativa no grupo A para a intensidade da dor na ATM do lado direito e na origem do masseter do lado esquerdo, sendo a maior intensidade da dor observada antes do uso da bandagem em ambas as regiões. Já para o grupo B, se observou diferença significativa na sensação de dor no temporal anterior do lado esquerdo, onde antes da intervenção obteve-se maior intensidade da dor.

**Tabela 2:** Comparação da intensidade da dor antes e após aplicação da bandagem elástica

Pontos de aferição da dor	Intensidade da dor									
	Grupo A (n=11)					Grupo B (n=11)				
	Antes		Após		Valor de p^1^	Antes		Após		Valor de p^1^
	Média	Desvio padrão	Média	Desvio padrão		Média	Desvio padrão	Média	Desvio padrão	
Temporal posterior – D	0,09	0,30	0,64	1,21	0,167	1,64	3,04	1,09	2,21	0,192
Temporal posterior – E	0,91	1,30	0,55	1,21	0,531	0,91	1,64	1,00	1,79	0,863
Temporal médio – D	1,27	2,15	1,18	1,72	0,831	1,91	2,59	1,18	1,60	0,104
Temporal médio – E	1,18	1,78	1,00	1,84	0,774	1,73	2,87	1,00	1,55	0,167
Temporal anterior – D	1,82	3,31	1,18	1,99	0,370	2,18	2,27	1,27	1,85	0,085
Temporal anterior – E	1,82	2,40	0,64	1,12	0,109	1,64	2,58	1,09	2,07	0,025*
ATM - D	1,64	2,50	0,45	1,04	**0,040** *	1,36	1,75	1,00	1,95	0,553
ATM - E	0,45	1,04	0,36	0,81	0,796	1,82	2,27	1,09	1,45	0,356
ATM aberta - D	1,73	2,94	1,55	2,84	0,881	1,27	2,28	1,00	2,14	0,192
ATM aberta - E	1,00	1,55	1,00	1,84	0,999	2,18	2,89	1,00	1,55	0,168
Masseter origem - D	2,45	3,21	2,18	2,75	0,661	2,73	2,57	1,82	1,83	0,157
Masseter origem - E	2,55	2,91	1,00	1,34	0,036*	2,18	2,64	1,55	1,75	0,361
Masseter médio - D	2,64	2,91	3,45	2,94	0,314	4,09	3,75	3,18	3,03	0,148
Masseter médio - E	3,00	2,68	2,82	3,19	0,859	4,27	3,32	3,45	3,42	0,268
Masseter inserção - D	2,82	2,56	2,45	2,98	0,623	4,27	3,00	2,73	2,97	0,068
Masseter inserção - E	3,64	3,17	3,36	3,11	0,493	4,18	2,99	3,36	3,17	0,213

* p<0,050

1 Teste *t* de *'Student'* para amostras pareadas

**Legenda:** ATM=articulação temporomandibular; D=direita; E=esquerda

A comparação da amplitude dos movimentos mandibulares nos momentos pré e pós-intervenção encontra-se na Tabela 3. Não houve diferença significativa entre as médias de amplitude de movimentos mandibulares entre os momentos antes e após intervenção em ambos os grupos.

**Tabela 3:** Comparação da amplitude dos movimentos mandibulares entre os momentos antes e após a aplicação da bandagem elástica

Movimentos mandibulares	Amplitude dos movimentos mandibulares									
	Grupo A (n=11)					Grupo B (n=11)				
	Antes (mm)		Após (mm)		Valor de p^1^	Antes (mm)		Após (mm)		Valor de p^1^
	Média	Desvio padrão	Média	Desvio padrão		Média	Desvio padrão	Média	Desvio padrão	
Abertura sem dor	47,13	6,89	46,88	7,55	0,811	46,14	9,06	46,32	11,37	0,953
Abertura de boca não assistida	54,15	5,38	53,49	5,14	0,151	54,04	7,82	50,96	12,80	0,286
Abertura de boca assistida	56,10	6,34	56,48	6,09	0,403	55,69	8,46	52,70	12,94	0,290
Lateralização para direita	8,70	2,09	9,17	1,79	0,307	8,26	2,80	8,14	3,72	0,795
Lateralização para esquerda	9,14	2,47	9,13	2,02	0,981	7,75	3,41	8,47	2,20	0,491
Protrusão	7,69	1,29	7,86	0,91	0,668	8,77	2,19	8,71	1,76	0,800

1 Teste *t* de *'Student'* para amostras pareadas

Não foi encontrada diferença significativa na redução da intensidade da dor ou na diferença da amplitude dos movimentos mandibulares entre os grupos (Tabela 4).

**Tabela 4:** Comparação da redução da intensidade da dor e da diferença de amplitude dos movimentos mandibulares entre os grupos após a aplicação da bandagem elástica

Variáveis dependentes	Variável independente	Grupo B	Erro Padrão	Valor p*	IC de 95% para Grupo B	
					Limite inferior	Limite superior
Temporal posterior D	Grupo A	-0,455	0,760	0,556	-2,039	1,130
Temporal posterior E	Grupo A	-0,455	0,652	0,494	-1,814	0,905
Temporal médio D	Grupo A	0,034	0,709	0,999	-1,479	1,589
Temporal médio E	Grupo A	0,012	0,726	0,999	-1,515	1,785
Temporal anterior D	Grupo A	-0,091	0,819	0,913	-1,800	1,618
Temporal anterior E	Grupo A	-0,455	0,710	0,529	-1,936	1,027
ATM - D	Grupo A	-0,545	0,666	0,422	-1,934	0,843
ATM - E	Grupo A	-0,727	0,500	0,161	-1,769	0,315
ATM aberta D	Grupo A	0,545	1,073	0,617	-1,694	2,784
ATM aberta E	Grupo A	0,100	0,726	0,999	-1,515	1,578
Masseter origem D	Grupo A	0,364	0,997	0,719	-1,715	2,443
Masseter origem E	Grupo A	-0,545	0,666	0,422	-1,934	0,843
Masseter médio D	Grupo A	0,273	1,273	0,833	-2,383	2,929
Masseter médio E	Grupo A	-0,636	1,409	0,656	-3,575	2,303
Masseter inserção D	Grupo A	-0,273	1,268	0,832	-2,918	2,373
Masseter inserção E	Grupo A	0,187	1,339	0,999	-2,792	2,988
Abertura sem dor	Grupo A	0,564	4,116	0,892	-8,021	9,149
Abertura não assistida	Grupo A	2,527	4,160	0,550	-6,150	11,204
Abertura assistida	Grupo A	3,782	4,311	0,391	-5,211	12,775
Lateralização para direita	Grupo A	1,021	1,245	0,422	-1,577	3,619
Lateralização para esquerda	Grupo A	0,655	0,900	0,475	-1,223	2,534
Protrusão	Grupo A	-0,845	0,597	0,172	-2,090	0,399

Nota: apenas dados após a aplicação da bandagem elástica

(*) Regressão linear simples

**Legenda:** B - Coeficiente; O grupo B é o de referência; significativo se p≤0,050; ATM = articulação temporomandibular; D - Direita; E – Esquerda; IC=intervalo de confiança; Grupo A com n=11 e Grupo B com n=11

A Tabela 5 apresenta a associação das sensações reportadas após o uso da bandagem entre os dois grupos. Não houve diferença significativa nas sensações reportadas entre os grupos, portanto, ter incômodo, coceira, sensação de relaxamento, dor ou de aumento da mobilidade independe de a bandagem ser aplicada sem estiramento ou com 40% de estiramento.

**Tabela 5:** Comparação das sensações reportadas após o uso da bandagem elástica entre os dois grupos

Sensações reportadas		Grupo A (n=11)		Grupo B (n=11)		Valor de p^1^
		N	%	n	%	
Sensação de incômodo	Não	7	63,64	7	63,64	0,999
	Sim	4	36,36	4	36,36	
Sensação de coceira	Não	9	81,82	10	90,91	0,999
	Sim	2	18,18	1	9,09	
Sensação de relaxamento	Não	6	54,55	7	63,64	0,999
	Sim	5	45,45	4	36,36	
Sensação de melhora da dor	Não	9	81,82	10	90,91	0,999
	Sim	2	18,18	1	9,09	
Sensação de melhora da mobilidade	Não	9	81,82	9	81,82	0,999
	Sim	2	18,18	2	18,18	

1 Teste Exato de Fisher

**Legenda:** n = número de participantes; % = frequência relativa de participantes

## DISCUSSÃO

A hipótese examinada no presente estudo pressupunha que o uso da bandagem sobre o masseter reduziria a dor e aumentaria a mobilidade mandibular e que a aplicação com estiramento teria os maiores efeitos. Os resultados encontrados não confirmam a hipótese do presente estudo, visto que não houve melhora de mobilidade e a diminuição na sensação de dor ocorreu em pontos isolados. Além disso, não houve diferença entre os grupos.

Não foram encontrados na literatura artigos que avaliaram os efeitos do uso da bandagem, de forma isolada, sobre o masseter em 24 horas. Um estudo investigou o uso da bandagem elástica com 40% de tensão, durante cinco semanas, em pacientes com bruxismo do sono e mostrou que, após uma semana, os participantes apresentaram diminuição da dor em músculos mastigatórios^(18)^. Outro estudo^(19)^ sugere benefícios do uso da bandagem elástica, por uma semana, com estiramento leve (0% a 15%) associado a orientações e exercícios mandibulares em pacientes com DTM na redução da dor à palpação em músculos masseter e temporal, bem como na região da articulação. Lietz-Kijak^(17)^ e sua equipe encontraram redução da intensidade da dor orofacial após 5 dias de uso da bandagem sobre masseter sem estiramento. Acredita-se que o curto tempo de uso da bandagem, nesta pesquisa, seja uma das razões para a pouca melhora dos sintomas dolorosos, bem como o fato de este recurso ter sido usado de forma isolada, sem outras intervenções, como aconselhamento, por exemplo.

Lemos^(13)^ elenca os benefícios do uso da bandagem elástica em quatro funções: dérmica, muscular, articular e linfática. Os efeitos, no caso da função dérmica, ocorrem em resposta à estimulação sensorial provocada pelo contato da fita com a pele, ativando mecanorreceptores cutâneos e, consequentemente, nervos periféricos por meio de tensões, pressões, descompressões e elevações da pele^(13)^. A estimulação dos mecanorreceptores que estão localizados na pele provoca a ativação de neurônios aferentes, cujos corpos situam-se nos gânglios dorsais da medula espinhal. O estímulo mecânico gerado pela bandagem concorre nas vias aferentes com o estímulo da dor, promovendo inibição deste^(12,13)^. Além disso, a bandagem, ao elevar a pele, promove descompressão de nociceptores pela redução do edema local^(13)^. Além do alívio da dor e do desconforto local e em tecidos subjacentes, a bandagem promove maior propriocepção dos movimentos e alinhamento articular. Quando aplicada com ponto fixo na inserção do músculo, a bandagem realiza, ainda, recessão da ação muscular, promovendo relaxamento dos músculos hiperfuncionais^(13)^.

Uma vez que a ação da bandagem ocorre, principalmente, a nível cutâneo, esperava-se maior redução da intensidade dolorosa no grupo A, de maior estiramento, portanto, maior intensidade do estímulo sensorial aplicado. Nesse sentido, os resultados da presente pesquisa vão ao encontro da hipótese, uma vez que o grupo A apresentou redução da dor em mais pontos de aferição, o que pode ter sido proporcionado devido ao maior estímulo sensorial aplicado, em comparação com o grupo B. Entretanto, o uso por apenas 24 horas pode não ter sido suficiente para um alívio da dor em maior quantidade de pontos. Outra possível explicação para não se ter encontrado diferença significativa na redução da dor foi o baixo nível de dor na avaliação inicial dos participantes da pesquisa, com média menor que 4,5 nos pontos avaliados, o que ofereceu pouca oportunidade de variação do nível de dor na análise final.

O uso da bandagem elástica no presente estudo não foi capaz de modificar a abertura de boca ou proporcionar maior lateralização e protrusão de mandíbula. Acredita-se que a ausência de exercícios de mobilidade mandibular, ou outras abordagens terapêuticas como terapia miofuncional, na intervenção realizada e, ainda, o tempo de uso de 24 horas podem ter influenciado tal resultado^(19)^. O estudo de Keskinruzgar e colaboradores^(18)^ constatou que a aplicação da bandagem elástica proporcionou aumento da abertura de boca, após cinco semanas, em pacientes com bruxismo, contudo, na mensuração realizada pelos autores na primeira semana de uso da bandagem, os resultados ainda não haviam indicado melhora significativa. Já no estudo de Benlidayi e equipe^(19)^, houve a realização de tratamento com bandagem associado aos exercícios de Fisioterapia durante seis semanas em 28 pacientes com DTM, verificando, após seis semanas, uma melhora na limitação funcional da abertura máxima de boca e aumento nos movimentos laterais direitos no grupo de estudo, os quais não podem ser atribuídos ao uso exclusivo da bandagem. No estudo de Ozmen e colaboradores^(23)^, os autores verificaram aumento da mobilidade mandibular no grupo que utilizou bandagem, porém, a bandagem também não foi aplicada de forma isolada, mas em conjunto com prescrição de medicamentos anti-inflamatórios e miorrelaxantes, placa oclusal e exercícios.

O presente estudo não encontrou diferença entre os grupos A e B tanto para dor quanto para amplitude de movimento com uso da bandagem por um dia. Esse resultado concorda com Emérito e colaboradores^(12)^, em sua revisão sistemática, que apontou que o uso da bandagem elástica em região do músculo masseter proporciona diminuição da dor após uma semana de tratamento. Especula-se que, nesse sentido, o maior tempo de tratamento poderia promover diferenças entre os grupos. Por não haver estudos que investigassem tais desfechos após 24 horas de uso da bandagem, considerou-se importante esta investigação e sugere-se que futuros estudos sejam realizados com diferentes estiramentos e durações de uso da bandagem.

O uso da bandagem com 40% de estiramento, no presente estudo, teve como referência o estudo de Keskinruzgar e colaboradores^(18)^ que obteve resultados positivos para dor e abertura de boca em indivíduos com bruxismo após o uso da bandagem com esta tensão. A aplicação da bandagem sem estiramento no presente estudo foi utilizada para compor um grupo controle com o objetivo de verificar o efeito do estiramento com 40% de tensão. Apesar da ausência de diferença significativa entre os grupos, ressalta-se que o alívio da dor foi percebido em mais pontos no grupo que fez uso da bandagem tensionada.

De acordo com os resultados obtidos, as sensações relatadas após o uso da bandagem não foram diferentes entre os grupos. Portanto, apresentar sensações de relaxamento, incômodo, coceira, dor ou de aumento da mobilidade, independe do estiramento da bandagem aplicado em 24 horas. A maior parte dos indivíduos de ambos os grupos relatou não notar relaxamento ou melhora da dor. Entretanto, a percepção do corpo é algo subjetivo e pode ser dependente de outros fatores como a história pessoal do sujeito^(16)^, assim como a sensação de redução da dor, que pode ter passado despercebido para alguns indivíduos^(24)^.

As limitações encontradas neste estudo foram o baixo número amostral, a heterogeneidade da amostra quanto ao diagnóstico da DTM, as perdas de participantes durante a coleta, o tempo reduzido de uso da bandagem e o fato de os indivíduos terem apresentado dor leve, com valores, no geral, menores que quatro. Sendo assim, os resultados desta pesquisa devem ser considerados com cautela e não generalizados. Como ponto forte, o presente estudo é inédito, sendo o primeiro no meio científico a investigar a interferência da bandagem elástica na dor e nos movimentos mandibulares com diferentes estiramentos, realizado de forma randomizada e duplamente cega.

## CONCLUSÃO

O uso da bandagem elástica por 24 horas sobre o músculo masseter com estiramento de 40% produziu, na população estudada, alívio da sensação de dor apenas na região da origem do músculo masseter esquerdo e na região da articulação temporomandibular direita, enquanto o uso sem estiramento diminuiu a dor em região do músculo temporal anterior esquerdo. Não houve diferença na amplitude de movimentos mandibulares após uso com ou sem estiramento da bandagem por 24 horas.
